# An important change in infective endocarditis prevention guidance

**DOI:** 10.1038/s41415-026-0005-0

**Published:** 2026-08-03

**Authors:** Martin H. Thornhill, Charlotte Eckhardt, Bernard Prendergast, Mark Dayer, Greg Hutton, Larry M. Baddour

**Affiliations:** 579098327192432423118 School of Clinical Dentistry, University of Sheffield, Sheffield, United Kingdom; 984531528382260043594 Maxillofacial Department, Morriston Hospital, Swansea Bay University Health Board, United Kingdom; 598480166707480733811 Cleveland Clinic, London, United Kingdom; 407884002850166982953 Cardiovascular Research Institute, Mater Private Network, Dublin, Ireland; Faculty of Health, University of Plymouth, Plymouth, United Kingdom; 506289445736896479633 Infective Endocarditis Patient, Patient Advocate, United Kingdom; 413069502284780388302 Division of Public Health, Infectious Diseases, and Occupational Health, Departments of Medicine and Cardiovascular Medicine, Mayo Clinic College of Medicine and Science, Rochester, Minnesota, USA

## Abstract

Recent changes to the National Institute for Health and Care Excellence (NICE) guidelines combined with an update to the implementation advice from the Scottish Dental Clinical Effectiveness Programme (SDCEP) have resulted in significant changes to the advice given to dentists concerning the prevention of infective endocarditis (IE). In contrast to a previous NICE recommendation against the use of AP for IE prevention, dentists are now recommended to offer antibiotic prophylaxis (AP) to all individuals at high-risk of IE before extractions or oral surgery procedures, and to consider AP for high-risk individuals undergoing any other at-risk dental procedures that involve manipulation of the gingival or periapical region of the teeth.

Most UK cardiologists, cardiothoracic surgeons and infectious disease specialists (alongside many National Health Service trusts, several of which incorporate dental schools and hospitals) follow the European Society for Cardiology (ESC) guidelines. Awareness of the changes made by NICE and SDCEP, and the similarities and differences between their updated recommendations and the ESC guidelines, is therefore essential.

**Supplementary Information:**

Zusatzmaterial online: Zu diesem Beitrag sind unter https://doi.org/10.1038/s41415-026-0005-0 für autorisierte Leser zusätzliche Dateien abrufbar.

## Key points

- There has been a large and important change in endocarditis prevention guidance for UK dentists.
- Dentists are now recommended to give antibiotic prophylaxis (AP) to all high-risk patients undergoing extractions and oral surgery procedures.
- Consider AP for all other at-risk dental procedures involving manipulation of the gingival or periapical region of the teeth.

**Supplementary Information:**

Zusatzmaterial online: Zu diesem Beitrag sind unter https://doi.org/10.1038/s41415-026-0005-0 für autorisierte Leser zusätzliche Dateien abrufbar.

## Background

Infective endocarditis (IE) is a serious bacterial infection of the heart valves, with a 30% mortality in the first year after diagnosis and significant ongoing morbidity among survivors.^1^ Approximately 30–40% of cases are caused by bacteria originating from the mouth^2^ and prevention is a primary focus of management in those at highest risk.

The British Society for Antimicrobial Chemotherapy (BSAC) recommended antibiotic prophylaxis (AP) for high-risk individuals undergoing invasive (at-risk) dental procedures.^3^ But, this changed in 2008 when the National Institute for Health and Care Excellence (NICE) issued new guidance that, ‘antibiotic prophylaxis against infective endocarditis is not recommended for people undergoing dental procedures'.^4^ Although their wording was softened in 2016 to ‘antibiotic prophylaxis against infective endocarditis is not recommended routinely for people undergoing dental procedures',^4^ the guidance against AP remained essentially unchanged.

In November 2024, however, NICE changed the wording of its guidelines once more and referred dentists, medical practitioners and patients to implementation advice produced by the Scottish Dental Clinical Effectiveness Programme (SDCEP): ‘antibiotic prophylaxis against infective endocarditis is not recommended routinely for people undergoing dental procedures. For advice on antibiotic prophylaxis for people at high risk of infective endocarditis undergoing dental procedures and for relevant patient information, see the SDCEP's implementation advice on antibiotic prophylaxis against infective endocarditis (this programme is part of National Health Service [NHS] Education for Scotland). It has been endorsed for use across the UK by the Chief Dental Officers of the UK'.^4^

Thereby – and for the first time – NICE acknowledged that some individuals are at high IE risk and could benefit from AP. Furthermore, it was the first time NICE had clearly directed dentists to use the implementation advice produced by SDCEP and confirmed that it applies to dentists across the UK (not just Scotland). The original SDCEP advice was based on the 2015 European Society for Cardiology (ESC) guidance but became outdated following the 2023 ESC update. Both NICE and SDCEP indicated that the implementation advice would be updated in line with the ESC changes, and their update was published on 24 March 2026.^5^ This update, combined with the change in NICE wording, transforms the guidance for IE prevention in the UK, which is now more closely aligned with the 2023 ESC recommendation that all high-risk individuals should receive AP before at-risk dental procedures.

Herein, we review the principal features of the new UK guidance and highlight similarities and differences with the ESC guidelines. These similarities and differences are important since most cardiologists and cardiothoracic surgeons follow the ESC guidelines. Moreover, in recent years, many NHS Hospital Trusts (including several which incorporate dental schools and hospitals) have aligned themselves with ESC guidelines.

## Main recommendation of the NICE/SDCEP advice

For patients at high-risk of IE, AP is recommended for extractions and oral surgery procedures and should be considered for all other at-risk dental procedures (Box 1).

Box 1 Principal recommendation of the NICE guidance and updated SDCEP implementation advice For patients at high-risk of infective endocarditis, antibiotic prophylaxis is recommended for extractions and oral surgery procedures and should be considered for all other at-risk dental procedures

### High-risk individuals

SDCEP has adopted the ESC definitions of high-risk patients (Box 2),^6^ and recommends cardiological consultation if there is any doubt about an individual's risk status.

Box 2 Individuals at high- and moderate-risk of infective endocarditis **High-risk patients**

Antibiotic prophylaxis is recommended in patients with:

- A previous episode of infective endocarditis
- Surgically implanted prosthetic valves and with any material used for surgical cardiac valve repair
- Transcatheter implanted aortic and pulmonary valve prostheses
- Untreated cyanotic congenital heart disease
- Congenital heart disease treated with surgery or transcatheter procedures with postoperative palliative shunts, conduits or other prostheses
- After surgical repair, in the absence of residual defects or valve prostheses, antibiotic prophylaxis is recommended only for the first six months after the procedure
- Ventricular assist devices.

Antibiotic prophylaxis should be considered in patients with:

- Transcatheter mitral and tricuspid valve repair.

Antibiotic prophylaxis may be considered in:

- Recipients of heart transplants.

**Moderate- or intermediate-risk patients**

Antibiotic prophylaxis not recommended routinely* in patients with:

- Rheumatic heart disease
- Non-rheumatic degenerative valve disease, e.g., mitral valve prolapse
- Congenital valve abnormalities including bicuspid aortic valve disease
- Hypertrophic cardiomyopathy
- Cardiovascular implanted electronic devices, e.g., implanted pacemakers and defibrillators.

Adapted from the 2023 European Society of Cardiology guidelines^6^ for the management of infective endocarditis and 2nd Edition of the Scottish Dental Clinical Effectiveness Programme implementation advice.^5^ *While not recommending antibiotic prophylaxis routinely for moderate-risk individuals, the European Society of Cardiology guidance states that antibiotic prophylaxis may be considered for an individual patient when a cardiologist feels this is appropriate, e.g., complex cardiac risk factors, or comorbidities such as diabetes or immune suppression.

### Moderate-risk individuals

Neither the ESC nor SDCEP recommend AP in patients with moderate IE risk (Box 2). However, the ESC guidelines state that AP may be considered in individual moderate-risk patients where a cardiologist feels this is appropriate (for example, in the presence of complex cardiac risk factors, or comorbidities such as diabetes or immune suppression). SDCEP support this view and say that such patients should be considered as high-risk if this is the cardiological recommendation.

### Low-risk individuals

The vast majority (>99%) of the population are at low risk and do not require AP for any procedure.

## At-risk dental procedures

Both the ESC and SDCEP use the term ‘at-risk' dental procedures (rather than ‘invasive dental procedures'), defined as: ‘at-risk dental procedures include dental extractions, oral surgery procedures (including periodontal surgery, implant surgery, and oral biopsies), and dental procedures involving manipulation of the gingival or periapical region of the teeth (including scaling and root canal procedures)'.^6^

Importantly, while the ESC recommends AP for all at-risk dental procedures, SDCEP considers ‘at-risk' dental procedures in two groups: i) extractions and oral surgery procedures – for which AP is recommended (Box 3); and ii) other at-risk dental procedures involving manipulation of the gingival or periapical region of the teeth – for which AP should be considered (Box 3).^5^ It is important that hospital-based specialists and primary-care dentists are aware of this difference since it is a potential source of ambiguity, misunderstanding and confusion (especially for patients). In particular, dentists should note that SDCEP now includes several new ‘at-risk' dental procedures that it previously excluded – including supragingival scale and polish, the basic periodontal exam and placement/removal of orthodontic separators and bands.^5^

Box 3 At-risk dental procedures where Scottish Dental Clinical Effectiveness Programme recommend antibiotic prophylaxis **At-risk dental procedures where antibiotic prophylaxis is recommended**

Extractions and oral surgery procedures including:

- Dental extractions
- Incision and drainage of abscess
- All oral surgical procedures
- Periodontal and endodontic surgery
- Placement of dental implants including temporary anchorage devices and mini implants
- Uncovering implants and implant components that are sub-mucosal
- Oral biopsies*.

**At-risk dental procedures where antibiotic prophylaxis should be considered**

Other procedures that involve manipulation of the gingival or periapical region of the teeth including:

- Professional mechanical plaque removal. This includes supra- and subgingival scaling
- Full periodontal examinations (including pocket charting)
- Basic periodontal examination
- Plaque and bleeding indices
- Subgingival restorations including fixed prosthodontics
- Placement of preformed metal crowns
- Placement of subgingival rubber dam clamps and subgingival matrix bands
- Placement and removal of orthodontic separators and bands
- Endodontic treatment before apical stop has been achieved.

Adapted from the 2023 European Society of Cardiology guidelines^6^ for the management of infective endocarditis and 2nd Edition of the Scottish Dental Clinical Effectiveness Programme implementation advice.^5^ *Although not specifically mentioned by the Scottish Dental Clinical Effectiveness Programme, oral biopsies are defined as an oral surgical procedure by the European Society of Cardiology guidelines.

## Antibiotic prophylaxis regimens

### Amoxicillin

The ESC guidelines recommend that high-risk patients receive a single 2 g dose of oral amoxicillin 30–60 minutes before at-risk dental procedures. Before 2008, UK guidelines recommended a single 3 g oral dose of amoxicillin in the form of a sugar-free powder sachet mixed with water that was highly effective, well-tolerated and reduced potential for antimicrobial resistance.^7^ However, this formulation was only available in the UK. In other countries, to give a 3g dose it was necessary to give six 500 mg capsules, which was associated with poor tolerance and compliance. Hence, most countries recommend a 2 g dose (four 500 mg amoxicillin capsules) which is associated with improved tolerance and acceptable bacteraemia reduction.^7^ The 3 g sachets remain available in the UK and though more expensive, are very familiar to many dentists and patients. Very sensibly, therefore, SDCEP recommend either option (Table 1).

**Table 1:** Oral antibiotic prophylaxis regimens

**Oral antibiotic prophylaxis regimens for high-risk individuals undergoing at-risk dental procedures**			
**Situation**	**Antibiotic**	**Single oral dose 30–60 minutes before procedure**	
		**Adults**	**Children**
No allergy to penicillin or ampicillin	Amoxicillin	2 g (4 × 500 mg capsules)	50 mg/kg (maximum dose 2 g)
		Or: 3 g (3 g sachet of sugar-free powder)	
Allergy to penicillin or ampicillin	Clarithromycin	500 mg	15 mg/kg (maximum dose 500 mg)
	Or: Azithromycin	500 mg	15 mg/kg (maximum dose 500 mg)
**Other amoxicillin alternatives recommended by European Society of Cardiology**			
Allergy to penicillin or ampicillin	Cephalexin^†,‡^	2 g	50 mg/kg (maximum dose 2 g)
	Or: Doxycycline	100 mg orally	<45 kg, 2.2 mg/kg >45 kg, 100 mg
Adapted from the 2023 European Society of Cardiology guidelines^6^ for the management of infective endocarditis and 2nd Edition of the Scottish Dental Clinical Effectiveness Programme implementation advice.^5^ †Or other first- or second-generation oral cephalosporin in equivalent dose ‡Cephalosporins should not be used in patients with a history of anaphylaxis, angio-oedema or urticaria after penicillin or ampicillin (due to the risk of cross-sensitivity). Note: clindamycin is no longer recommended as antibiotic prophylaxis for a dental procedure.			

### Alternatives for those with a history of penicillin allergy

Previous guidelines recommended clindamycin for those with a history of penicillin allergy. However, several studies have demonstrated a significant risk of fatal and non-fatal adverse events (particularly *Clostridioides difficile* infections) after a single 600 mg dose,^8^ and both the ESC and SDCEP now recommend a single 500 mg oral dose of clarithromycin or azithromycin (or other alternatives) 30–60 minutes before a procedure (Table 1).

### Parenteral AP regimens

Parenteral AP regimens are generally only required in secondary care settings and full details are provided in the current ESC guidance and SDCEP implementation advice.^5^^,^^6^

## Advice for patients

### Risks, benefits and informed consent

**Fig. 1:**
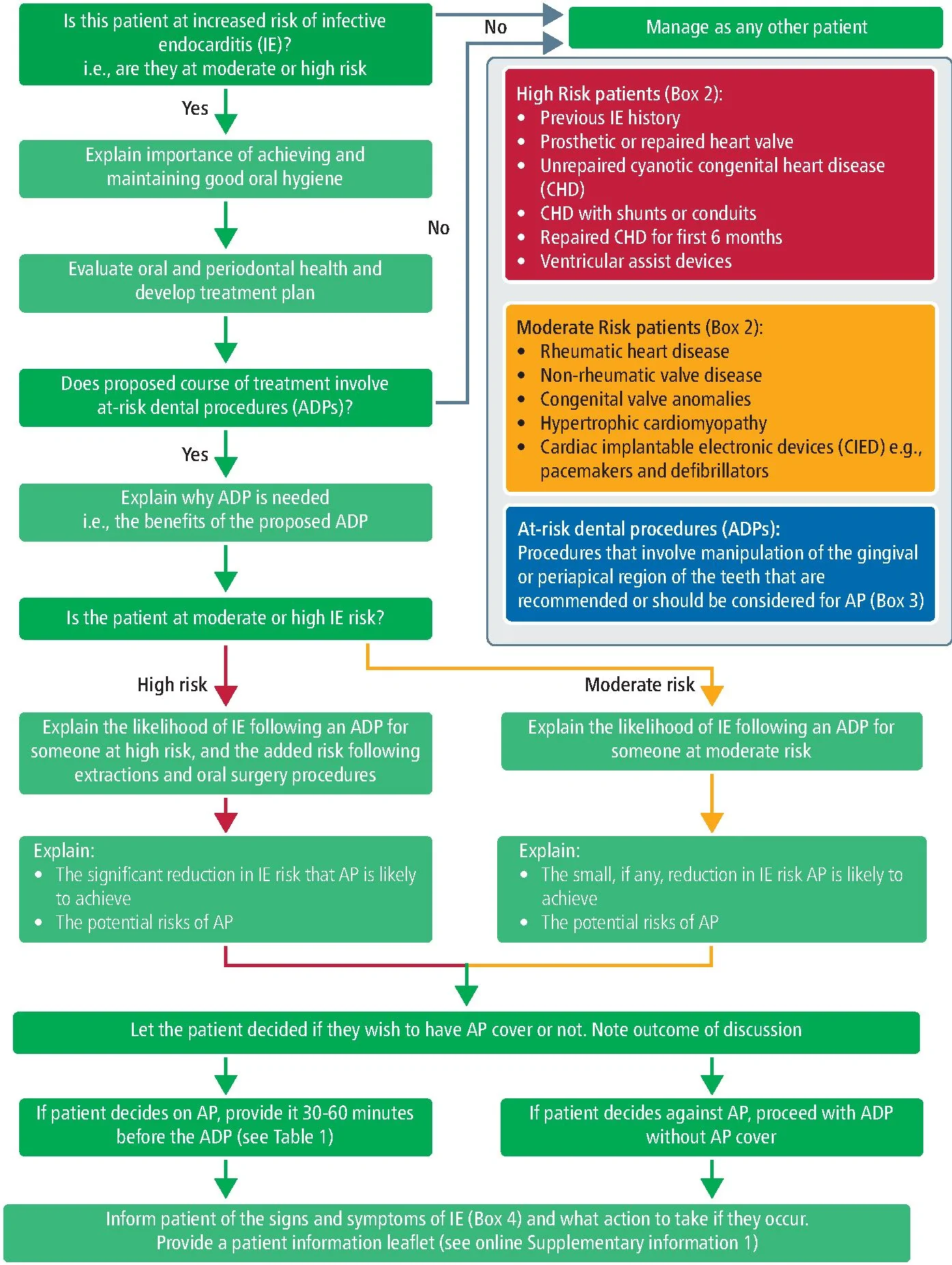
Flow chart for the management of antibiotic prophylaxis (AP) in patients at high- and moderate-risk of infective endocarditis

Guideline committees stress the importance of discussions concerning the risks and benefits of any proposed dental treatment with patients at increased IE-risk (both moderate and high) within the framework of informed consent, alongside the corresponding risks and benefits of AP. The content of these discussions and subsequent patient decision should then be recorded in the clinical notes. We have previously published background information to guide these discussions,^8^ and Figure 1 provides a flow chart to direct the management of patients at increased IE risk.

### General prevention measures

The 2023 ESC guidelines highlight the need for patients at increased risk to maintain good oral hygiene, recommending that, ‘patients should be encouraged to maintain twice daily tooth cleaning and to seek professional dental cleaning and follow-up at least twice yearly for high-risk patients and yearly for others'.^6^ SDCEP also notes the importance of oral hygiene but provides less specific advice. The ESC also highlights the importance of ‘curative antibiotics for any focus of bacterial infection' and ‘strict infection control measures for any at-risk procedure,' while also advocating ‘discouragement of piercing and tattooing', ‘no self-medication with antibiotics', ‘strict cutaneous hygiene, including optimised treatment of chronic skin conditions' and ‘disinfection of wounds'.^6^

### Recognition of IE

The possibility of IE is not eliminated by AP and all moderate- and high-risk patients who have undergone at-risk dental procedures (with or without AP) should be aware of symptoms suggesting the diagnosis (Box 4) and the actions needed should they arise. Although symptoms may arise soon after the procedure, they may be delayed for weeks. Patients should therefore be instructed to seek prompt medical assessment and raise the possibility of IE with their general practitioner since early diagnosis substantially improves clinical outcomes. Patient information leaflets have been provided by the *British Dental Journal* and SDCEP,^5^^,^^8^ and an updated version is available (see online Supplementary Information 1).

Box 4 Symptoms of infective endocarditis **Symptoms**

- High temperature (38°C or above)
- Night sweats
- Shortness of breath on exertion
- Tiredness (fatigue)
- Muscle and joint pains
- Unexplained weight loss
- Loss of appetite.

**Other presentations**

- New or changing heart murmur
- Spotty red skin rash (petechiae)
- Narrow, reddish-brown streaks under the nails (splinter haemorrhages)
- Red tender lesions on the fingers or toes (Osler's nodes)
- Confusion
- Stroke.

## Discussion

Before 2006, BSAC provided guidelines for IE prevention in the UK and recommended use of AP before invasive dental procedures for a large number of patients at moderate or high IE risk. In 2006, however, BSAC reviewed their guidelines and recommended restricting AP to high-risk individuals only, becoming the first guideline committee in the world to do so.^3^ This shocked the UK cardiology community, whose protests^3^^,^^9^^,^^10^ led the Department of Health to suspend the 2006 BSAC guidance and direct NICE to undertake a review. This concluded in April 2008, that ‘antibiotic prophylaxis against infective endocarditis is not recommended for people undergoing dental procedures'.^4^ As a consequence, the UK was isolated in recommending against AP, while other international guideline committees, including those of the American Heart Association in 2007 and the ESC in 2009,^11^^,^^12^ recommend limiting AP to high-risk individuals undergoing invasive dental procedures, as BSAC had recommended in 2006.

The Chief Dental Officers (CDOs) of England, Scotland, Wales and Northern Ireland wrote to every dentist in April 2008 advising them to comply with the NICE guidance and noting that for dentists in England and Wales, compliance was a condition of their NHS general service agreements. Meanwhile indemnity insurance providers informed dentists that they would not be covered if a patient developed an adverse drug reaction after use of AP. Not surprisingly, compliance with the new guidance was high.^13^^,^^14^

In 2016, NICE changed the wording of its guidance without consultation or review, to ‘antibiotic prophylaxis against infective endocarditis is not recommended routinely for people undergoing dental procedures'.^4^ However, the subtle addition of the word ‘routinely' caused enormous confusion by implying that there were non-routine situations when AP might be appropriate. But NICE provided no information about which patients or procedures might require AP – or which AP regimen should be used.

Importantly, NICE guidelines do not apply in Scotland where cardiologists largely follow the ESC guidelines. Although the CDO for Scotland advised Scottish dentists to adhere to NICE guidelines in 2008, they were not required to do so in the same way as their counterparts in England, Wales and Northern Ireland. Consequently, following insertion of the word ‘routinely' into the NICE guidelines, SDCEP produced advice for dentists on the implementation of the ambiguous NICE guidance in 2018 that was based upon the 2015 ESC guidelines (recommending that AP be considered for all high-risk individuals). The subsequent decision by NICE to endorse this SDCEP advice was not clearly stated within the NICE guidelines or communicated to dentists. Consequently, most dentists in England and Wales continued to follow earlier NICE guidelines avoiding the use of AP.

The ESC reviewed their guidelines once more in 2023 in the light of accumulating evidence concerning the safety and efficacy of AP,^2^ and increased the strength of their recommendation supporting its use in all high-risk individuals from Grade IIa (AP ‘should be considered') to Grade I (AP is ‘recommended').^6^ This stimulated NICE to undertake a surveillance review in June 2024 that concluded that a full review was not warranted. Nevertheless, in November 2024, NICE changed the wording of its guidelines (again without consultation or review), as follows: ‘antibiotic prophylaxis against infective endocarditis is not recommended routinely for people undergoing dental procedures. For advice on antibiotic prophylaxis for people at high risk of infective endocarditis undergoing dental procedures and for relevant patient information, see the SDCEP's implementation advice on antibiotic prophylaxis against infective endocarditis (this programme is part of NHS Education for Scotland). It has been endorsed for use across the UK by the CDOs of the UK*.*'

This was the first time, therefore, that NICE acknowledged that some individuals are at high risk of IE and could benefit from AP. Also, for the first time, NICE has clearly directed all UK dentists to use the SDCEP advice. The SDCEP advice was out of date, however, prompting NICE and SDCEP to immediately announce that it would be updated to take account of the 2023 ESC guideline changes. The updated second edition of the SDCEP implementation advice was published on 24 March 2026.^5^

### The importance of this change

These updated recommendations from NICE and SDCEP represent a fundamental change for UK dentists with major implications for everyday clinical practice.

For the first time in 18 years, UK dentists are again recommended to provide (or consider providing) AP to high-risk patients undergoing at-risk dental procedures. Ironically, BSAC was the first guideline committee in the world to make this recommendation in 2006,^3^ and it has taken 20 years for us to return to that position. During that time, many people have unnecessarily developed IE and some may have died as a result. It is important that we acknowledge the enormous loss that has potentially been caused to affected individuals and their families, as well as the associated financial cost.^15^

It is regrettable that NICE did not grasp the opportunity to unify IE prevention guidance across the UK, and Europe, by adopting or endorsing the ESC guidance. Having endorsed the SDCEP advice instead, it is a pity SDCEP did not more closely follow the ESC guidance. SDCEP is the only advisory body to have split dental procedures into two categories with different recommendations. Recommending AP for extractions and oral surgery procedures but not for other at-risk dental procedures could be misinterpreted as implying that other at-risk dental procedures pose a much lower risk than extractions and oral surgery procedures, and are therefore not worthy of AP.

Clearly, this is not what SDCEP intend since they say AP should be considered for these other at-risk procedures. But the rationale for ‘recommending' AP for extractions and oral surgery procedures but only suggesting it should ‘be considered' for other at-risk procedures is not obvious and is open to misinterpretation, ambiguity and confusion. While broadly welcoming the new SDCEP advice, we therefore contest that it would have been preferable to maintain concordance with the ESC guidelines and recommend AP for high-risk patients undergoing all at-risk dental procedures.^6^

Irrespective of these remaining points of debate, dentists should now discuss the risks of IE posed by any at-risk dental procedure with their patients and the risks and benefits of AP cover. They should also be aware that a recent systematic review demonstrated a significant risk of bacteraemia following all at-risk dental procedures,^16^ and that a study of 53.6 million at-risk dental procedures found an IE risk that was ~126 times greater for high-risk individuals than the general population following all at-risk dental procedures (OR 126.3; 95% CI 113.5–140.6; *p* <0.001).^17^ Although this risk was higher following extractions and oral surgery procedures, it was also very high for all other at-risk dental procedures. Finally, they should refer to two large clinical trials and a systematic review showing that AP reduces IE incidence following at-risk dental procedures in high-risk individuals.^2^^,^^18^^,^^19^ The case for providing AP cover to high-risk patients undergoing at-risk dental procedures is therefore based on strong clinical and scientific evidence.

## Conclusions and call for action

The combined impact of the change in NICE guidance and subsequent SDCEP update is welcome, and dentists are once more being advised to recommend AP for high-risk patients undergoing extractions and oral surgery procedures and to consider it for all other at-risk dental procedures.

This message needs to be urgently transmitted to all dentists and, as in 2008, the CDOs should write to every dentist to inform them of this important change. A synopsis of this guidance and recommended antibiotic regimens should be reinstated in the British National Formulary. Indemnity insurers should also update their indemnity cover arrangements and advise their members accordingly. Moreover, these topics should be urgently re-incorporated in the training curricula for dentists, dental hygienists, dental therapists and dental nurses. IE is a dangerous and life-threatening disease – and can be prevented by good holistic dental care and appropriate use of AP.

## Supplementary Information


Online Supplementary Information 1. Heart image courtesy of iStock, credit: sajithsaam (PDF 292KB)

