# Supplementary material for: An important change in infective endocarditis prevention guidance
Source: Br Dent J. 2026 Aug 3;241(4):261–6. doi: 10.1038/s41415-026-0005-0 (PMC13524779; doi:10.1038/s41415-026-0005-0)
Supplement: Supplementary file 1 [file 41415_2026_5_MOESM1_ESM.pdf]

# Infective endocarditis and dental treatment

## A patient information leaflet

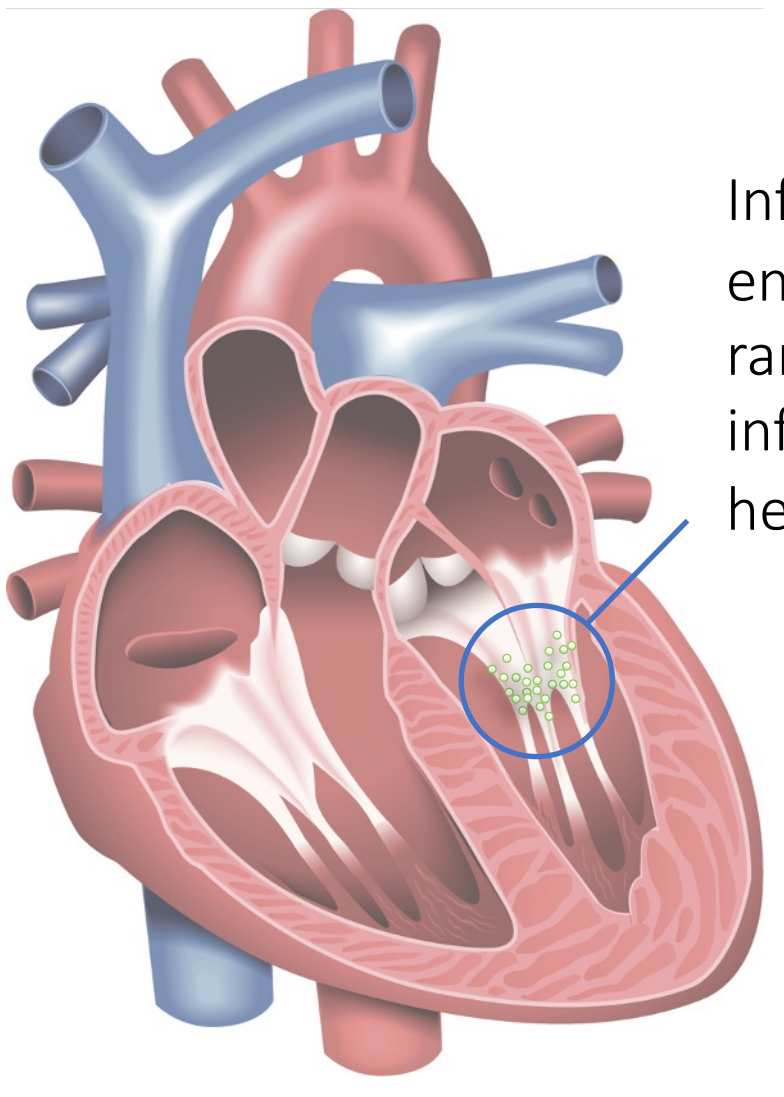

Infective endocarditis is a rare but serious infection of the heart valves

It can affect anyone, but you are at increased risk of endocarditis if you have one of the following:

**Higher-risk conditions:**

- A replacement heart valve.
- A history of endocarditis.
- Some types of congenital heart disease.
- Other high-risk conditions advised by your cardiologist

**Moderate-risk conditions:**

- Unoperated heart valve disease.
- A leaking or narrowed heart valve.
- An implanted pacemaker or defibrillator.
- Hypertrophic cardiomyopathy.

If you have a high or moderate risk condition, there is a small risk of developing endocarditis after at-risk dental procedures.

**At-risk dental procedures** include:

- Oral surgery procedures (all types).
- Tooth extractions.
- Any procedure involving manipulation of the gums or periapical region (root tips) of the teeth (including scaling, root canal treatments and some restorative dental treatments).

If you need an invasive dental procedure your dentists will explain the risk and what can be done to prevent or minimise this.

## Endocarditis prevention:

### Oral Hygiene

- Maintenance of good oral hygiene is important in preventing endocarditis in those with moderate- and higher-risk conditions.
- Your dentists or hygienist will explain how this can be achieved.
- Patients with moderate-risk conditions should see their dentist or hygienist at least annually for professional cleaning and a dental exam
- Patients with higher-risk conditions should do so twice yearly

### Antibiotic prophylaxis

- Your dentist will explain the potential risks and benefits of taking an antibiotic before an invasive dental procedure to reduce the subsequent risk of endocarditis (antibiotic prophylaxis).
- It can significantly reduce the endocarditis risk for patients with higher-risk conditions.
- The benefit for those with moderate-risk conditions is much smaller (but so is the risk) and the benefits may not outweigh the disadvantages.
- After explaining the risks and benefits your dentist will let you decide if you wish to have antibiotic prophylaxis or not.

If you have a moderate- or higher-risk condition and have an at-risk dental procedure (even with antibiotic prophylaxis) you should be aware of the way endocarditis can present so that you can seek treatment promptly if needed. Early diagnosis and treatment has better outcomes.

## Endocarditis

### Presentation:

- Features can present acutely, soon after the dental treatment, or be delayed (sub-acute).
- It most commonly presents as a flu-like illness.
- It is difficult to diagnose because the symptoms are like flu or other similar conditions.

**Features** (that may come and go or not all be present)**:**

- **Fever** (temperature of 38°C or above).
- **Breathlessness.**
- **Tiredness** (fatigue).
- **Muscle and joint pains.**
- **Sweats or chills** (especially at night).
- **Unexplained weight loss.**
- Other possible features: rash, swollen lymph nodes, headache, nausea or poor appetite.

If features of endocarditis develop within a few weeks of an at-risk dental procedure

## What action to take if features of endocarditis develop within a few weeks of an at-risk dental procedure:

- **Seek urgent medical attention.**
- **Tell the doctor you are worried about endocarditis and what higher-risk or moderate-risk conditions you have.**
- **Inform the doctor about any invasive dental procedure you had** (and when).
- **Tell them if you had antibiotic prophylaxis or not before the procedure.**
- Also tell them if you had any other medical or surgical procedures, skin wounds, tattooing, body piercing, intravenous lines or intravenous drug use in the previous few weeks, since these can also predispose you to endocarditis.
